# Live Influenza Vaccine Provides Early Protection against Homologous and Heterologous Influenza and May Prevent Post-Influenza Pneumococcal Infections in Mice

**DOI:** 10.3390/microorganisms10061150

**Published:** 2022-06-02

**Authors:** Yulia Desheva, Galina Leontieva, Tatiana Kramskaya, Igor Losev, Andrey Rekstin, Nadezhda Petkova, Polina Kudar, Alexander Suvorov

**Affiliations:** Scientific and Educational Center “Molecular Bases of Interaction of Microorganisms and Human” of the World-Class Research Center “Center for Personalized Medicine”, Federal State Budgetary Scientific Institution “Institute of Experimental Medicine”, 12 Academician Pavlov Street, 197376 Saint Petersburg, Russia; galeonte@yandex.ru (G.L.); tatyana.kramskaya@gmail.com (T.K.); iemlosev@gmail.com (I.L.); arekstin@yandex.ru (A.R.); pn.nadezhda@yandex.ru (N.P.); polina6226@mail.ru (P.K.); alexander_suvorov1@hotmail.com (A.S.)

**Keywords:** live influenza vaccine, early cytokines, influenza infection, *Streptococcus pneumoniae*

## Abstract

Influenza and *S. pneumoniae* infections are a significant cause of morbidity and mortality worldwide. Intranasal live influenza vaccine (LAIV) may prevent influenza-related bacterial complications. The objectives of the study are to estimate resistance against early influenza infection and post-influenza pneumococcal pneumonia after LAIV in mice. Mice were administered intranasally the monovalent LAIV A/17/Mallard Netherlands/00/95(H7N3), A/17/South Africa/2013/01(H1N1)pdm09 or trivalent LAIV 2017–2018 years of formulation containing A/17/New York/15/5364(H1N1)pdm09 vaccine strain. LAIV demonstrated early protection against homologous and heterologous infections with A/South Africa/3626/2013 (H1N1) pdm09 influenza virus on day six, following immunization. Following boost immunization, trivalent LAIV demonstrated a pronounced protective effect both in terms of lethality and pneumococcal lung infection when *S. pneumoniae* infection was performed three days after the onset of influenza infection. Conclusion: LAIV provides early protection against homologous and heterologous viral infections and has a protective effect against post-influenza pneumococcal infection. These data suggest that the intranasal administration of LAIV may be useful during the cycle of circulation not only of influenza viruses, but also of other causative agents of acute respiratory infections.

## 1. Introduction

The best strategy and the only science-based way to effectively prevent influenza infection is immunization with modern influenza vaccines. The current epidemic situation, which is characterized by the onset of the antigenic drift of pandemic and potentially pandemic influenza viruses and an increase in the number of pneumococcal serotypes that differ from the serotypes of existing polysaccharide vaccines, requires the development and testing of vaccines that may protect against influenza and bacterial complications of influenza infection. The development of mucosal vaccines is urgently needed for specific prophylaxis of respiratory infections, as immunization with these vaccines provides the formation not only of systemic, but also local, immunity and makes it possible to acquire resistance to reinfection in a short period of time. Live attenuated influenza vaccine (LAIV) has been shown to be an effective influenza vaccine that is easy to administer, cost-efficient, and quick to produce [1]. LAIV is licensed and has been used in Russia for 50 years [1]. LAIV was licensed in the USA for healthy people from 2 to 49 years old in 2003, in Canada for patients from 2 to 59 years old since 2010, and in Europe for patients from 2 to 17 years old since 2011 [2]. LAIV has been approved in childhood vaccination programs in the UK since 2013 and Finland since 2015. Recently, the Russian LAIV has been licensed in China (http://www.biodiem.com/wp-content/uploads/2020/02/20-02-28-LAIV-influenza-vaccine-approved-in-China.pdf, assessed on 28 February 2020). The use of LAIV has potential advantages over traditional parenteral inactivated vaccines, especially in children—a long-term effect that provides an immune response that mimics natural infection [3]. Despite the official recommendations and vaccinations against seasonal influenza, vaccination rates for LAIV in age groups differ depending on the recommendations of the local health authorities in the country [4]. Vaccine strains of LAIV are obtained on the basis of attenuation donors adapted to cold (cold-adapted, *ca*-) and sensitive to elevated temperatures (temperature-sensitive, *ts*-). The effectiveness of LAIV is about 80% in children aged 6 years old and younger and about 40% in adults. LAIV is administered intranasally and can induce a longer, broader immune (humoral and cellular) response [5,6,7].

Activation of the conditionally pathogenic bacteria of the respiratory tract due to the action of the influenza virus on the barrier function of the epithelium leads to the occurrence of bacterial complications, the most common of which are bronchitis and bacterial pneumonia. A considerable amount of data was obtained from studying the effect of primary influenza infection on secondary bacterial invasion. The development of lethal synergism has been demonstrated for cases in which a bacterial infection occurs during the peak in viral reproduction [8]. At the same time, virus–bacterial antagonism effects have been demonstrated as well, for example, for the causative agent of porcine streptococcal infection (*Streptococcus suis*) and the A/H1N1 influenza virus, due to a direct interaction between hemagglutinin (HA), which blocks α2,6-linked sialic acids expressed on the bacterial membrane [9].

It has been shown that bacterial complications of influenza infection during the 2009 pandemic caused by A/H1N1 viruses were of the same significance as during the 2018 pandemic [10]. Since then, numerous clinical and laboratory studies have shown that infection with influenza viruses disrupts the signaling pathways of innate and adaptive immunity and reduces resistance to tissue damage, which increases susceptibility to bacterial infection. This leads to an increased proliferation of bacteria in the upper and lower respiratory tracts and increases the severity of bacterial complications, such as secondary bacterial pneumonia and otitis media [11]. In a mouse model of parenteral immunization with pseudoviruses containing HA of A/Puerto Rico/8/1934, it was shown that both vaccine-matching and non-matching HA antibodies may reduce morbidity and mortality due to post-influenza pneumococcal infections [12].

In respect to the widespread use of LAIV, the question arises of how the intranasal administration of LAIV affects influenza infection and bacterial contamination of the respiratory tract during the first days after immunization. There is evidence that the influenza vaccine prevented secondary bacterial infections in humans, while a reduced incidence of otitis media was observed in pediatric populations during clinical trials of the LAIV vaccine [13]. There are results that suggest that LAIV can provide non-specific protection against respiratory syncytial virus (RSV) infection, and this has been shown to be associated with a change in the cytokine profile upon the RSV challenge of immunized mice [14].

The objectives of this work are to demonstrate the early protection against influenza infection after the immunization of mice with LAIV of a potentially pandemic and pandemic subtype, and to demonstrate the protective efficacy of two doses of seasonal trivalent LAIV against pneumococcal post-influenza infection.

## 2. Materials and Methods

### 2.1. Ethics Statement

All procedures involving animals were performed according to the “Rules of Laboratory Practice” of the Ministry of Health of the Russian Federation No. 708 n. The study was approved by the Local Ethics Committee for Animal Care and Use at the”, Federal State Budgetary Scientific Institution “Institute of Experimental Medicine”, Saint Petersburg, Russia, Protocol No. 1/21, 28 January 2021.

The 8-to-10-week-old female CBA mice were acquired from the laboratory breeding nursery of the Russian Academy of Sciences (Rappolovo, Leningrad Region). Mice were maintained under standard conditions and given ten days to acclimate to the housing facility. Feeding was conducted ad libitum, in the morning with free access to water.

Non-terminal procedures were performed under ether anesthesia. No animal showed any signs of illness following vaccine strain inoculation. No animals died as a result of the vaccination procedures. To control viral and bacterial loads in the lungs, animals were euthanized under ether anesthesia and cervical dislocation. The health status of the the infected mice was monitored and recorded once a day for ten days post final vaccination, viral infection or bacterial superinfection.

The animals were immediately euthanized by cervical dislocation following ether anesthesia if they displayed abnormal behavior (desire to be alone), ruffled fur, reduced mobility, or hunchbacked posture. All efforts were made to minimize the suffering of the animals. No animals died before they met the euthanasia criteria.

### 2.2. Influenza Viruses

The vaccine viruses presented in Table 1 were used for the intranasal immunization of 8 to-10-week-old female Balb/c mice.

For challenging the immunized mice, we used pandemic influenza virus A/South Africa/3626/2013 (H1N1) pdm09. All viruses were propagated in chicken embryos (CEs), aliquoted, and stored at −70 °C before use. All viruses were obtained from the collection of the Federal State Budget Scientific Institution “Institute of Experimental Medicine”.

### 2.3. Infectious Pneumococci

*S. pneumoniae* clinical isolates serotype 3 strain 73 were grown anaerobically at 37 °C for 18 h in THB medium containing 20% horse serum (Difco, Carrickmore, UK). Columbia agar containing 5% defibrinated sheep blood and 10% horse serum was used as a dense medium for culturing and counting the number of bacteria.

### 2.4. Immunization and Sample Collection

Randomly determined groups of animals (25 mice per group) were lightly ether anesthetized and immunized intranasally (i.n.) with 50 µL divided equally per nostril. We used LAIV containing 1 × 10^7^ 50% embryonic infectious dose (EID_50_) of each vaccine strain. Animals in the control group were inoculated by PBS in the same manner. The second vaccination was performed with an interval of 21 days. Serum samples were collected from 6 mice from each vaccine group for the determination of HA-specific antibodies by ELISA. Saliva/nasal samples were collected from 6 mice from each group following intraperitoneal injection of 0.1 mL of 0.5% pilocarpine solution into tubes containing 0.001 M serine protease inhibitor-inhibitor phenylmethylsulfonyl fluoride (PMSF). Different investigators were responsible for sampling, analysis, and statistical processing.

### 2.5. Immunogenicity

Levels of serum IgG and local IgA were determined by ELISA in 96-well ELISA plates (Sarstedt, Nümbrecht, Germany) sensitized with whole purified virus A/South Africa/3626/13(H1N1)pdm09 (20 hemagglutination units, HAU) at 0.1 mL overnight at 4 °C, as previously described [15], with an initial dilution of 1:10. The final ELISA titers were expressed as the maximum dilution at which the optical density at 450 nm (OD450) exceeded the mean OD450 plus 3 standard deviations of the negative-control wells.

### 2.6. Early Protection against Influenza Infection

Challenge was performed on the 6th day after immunization using the following pandemic influenza viruses: A/New York/61/15 (H1N1)pdm09 or A/South Africa/3626/2013 (H1N1) pdm09 at infectious doses of 10 50% mouse lethal doses (MID50). To determine the viral titer in the lungs, the samples from 5 animals per group were grinded in PBS containing 100 U/mL penicillin, 100 μg/mL streptomycin, followed by centrifugation for 10 min at 6000× *g*. To determine the virus titer, the samples were titrated in CE using serial dilutions starting from 1:10. Virus titers were expressed as log10 EID50, as previously described [15]. Survival was observed within two weeks after the onset of viral challenge. The change in weight was expressed as a percentage, with the weight of the animals before infection taken as 100%.

### 2.7. Early Cytokine Production In Vitro

We used a human monocyte-macrophage cell line (THP-1) to evaluate early cytokine production in vitro. THP-1 cells were cultivated in 24-well tissue culture plates at 3.0 × 106 cells per well with RPMI (Roswell Park Memorial Institute, Buffalo, NY, USA) medium containing 10% fetal calf serum, 100 IU/mL of penicillin, and 100 μg/mL of streptomycin. The cells were incubated at 37 °C and 5% CO_2_ for 48 h before the experiment. Cells were inoculated with 10^6^ EID50/mL of A/17/Mallard Netherlands/00/95(H7N3) or A/17/South Africa/2013/01(H1N1)pdm09 LAIV viruses and “wild-type” parental strains: A/Mallard Netherlands/12/2000(H7N3) influenza virus or A/South Africa/3626/2013(H1N1)pdm09. As a positive control, we used a Toll-like receptor (TLR) agonist, polyinosinic:polycytidylic acid (Poly I:C) (Sigma, Saint Louis, MO, USA) in the final concentration of 1 µg/mL. Wells containing only RPMI medium were used as negative controls. The culture plates were further incubated for 24 h in RPMI. Cell supernatants were harvested at 6 h for ELISA cytokine assays. The levels of early cytokines (TNF-α, IL-6) and type-1 interferon (IFN-α) in supernatants were determined using commercial ELISA test systems provided by eBioscience (San Diego, CA, USA), according to the instructions of the manufacturer.

### 2.8. Post-Influenza Pneumococcal Pneumonia in Mice

On day 21, following the 2nd vaccine dose, the mice were challenged with 1 LD50 of pandemic influenza virus A/South Africa/3626/2013 (H1N1)pdm09. Pneumococcal super-infection was performed 24 h after viral infection using 5 × 10^4^ colony forming units (CFUs) of *S. pneumoniae*. The change in weight was expressed as a percentage, with the weight of the animals before infection taken as 100%. A total of 48 h after bacterial superinfection, the lungs from 5 mice per group were extracted and homogenized in PBS through a Retsch MM-400 ball vibratory mill (Retsch, Haan, Germany). To determine the virus titer, the samples were titrated in CE starting from a dilution of 1:10 as described above. Serial 10-fold dilutions of homogenates were seeded on a dense nutrient medium, “Columbia” agar containing 5% defibrinated sheep blood and supplied by 10% horse serum. The plates were incubated at 37 °C in 5% CO_2_ for 14–16 h until the colonies were counted under a microscope. The bacterial content in CFUs per organ was calculated as previously described [16] and expressed as log10.

To assess bacteremia, blood samples were obtained from the submandibular vein of mice on day 5 following bacterial infection, and the bacterial content in the blood was assessed on “Columbia” agar containing 5% defibrinated sheep blood and 10% horse serum as described above.

### 2.9. Statistical Analysis

Statistical data processing was performed using Prism 8 (GraphPad Software, San Diego, CA, USA). Means and standard deviations of means (SDs) were calculated to express viral or bacterial titers. Comparisons of two independent groups were conducted using the Mann–Whitney test. For categorical data, Fisher’s exact test was used. We used log-rank (Mantel–Cox test) to compare the survival distributions within the vaccinated groups of mice. The *p*-value < 0.05 was considered to be statistically significant.

## 3. Results

### 3.1. Study of Early Protection against Homologous and Heterologous Influenza Infections after A/Mallard Netherlands/12/2000(H7N3) LAIV Immunization

Since the infection of mice with the homologous A/Mallard Netherlands/12/2000(H7N3) influenza virus was not lethal [17], we conducted the infection of immunized mice with the pandemic A/South Africa/3626/2013 (H1N1)pdm09 influenza virus, capable of causing a lethal infection in mice, as previously shown [18].

**Figure 1:**
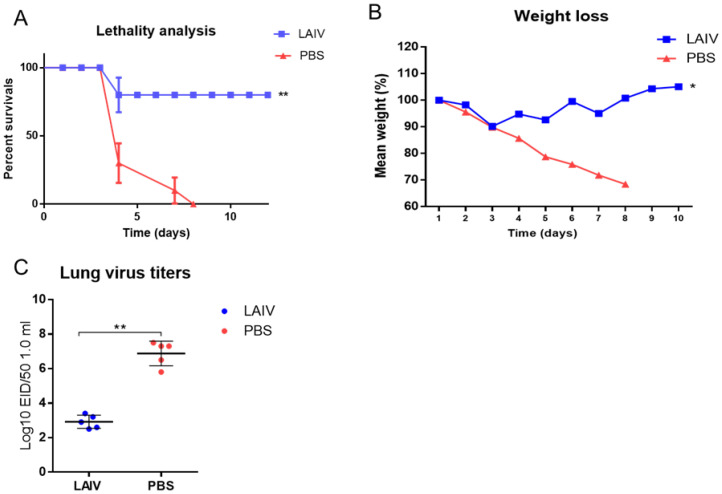
Challenge using 10 MLD50 of A/South Africa/3626/13(H1N1)pdm09 influenza virus on 6th day, following A/17/Mallard Netherlands/00/95(H7N3) immunization. * *p* < 0.05, ** *p* < 0.01 compared to PBS group. (**A**) Survival proportions (*n* = 10 per group). (**B**) Weight loss (*n* = 10). (**C**) Challenge-virus isolation from the lungs, 48 h after virus challenge (*n* = 5).

To assess the early protection following immunization with LAIV, a challenge with the A/South Africa/3626/2013 (H1N1)pdm09 influenza virus was performed on the 6th day after immunization. The results are presented in Figure 1.

As can be observed in Figure 1A, the immunization of mice with LAIV of a potentially pandemic subtype A/H7N3 protected 80% of mice from lethality following infection with the A/South Africa/3626/2013(H1N1)pdm09 influenza virus. This was accompanied by a decrease in weight loss, which amounted to no more than 10% (Figure 1B) and a decrease in the levels of infectious virus in the lungs (Figure 1C) in the group of vaccinated mice.

**Figure 2:**
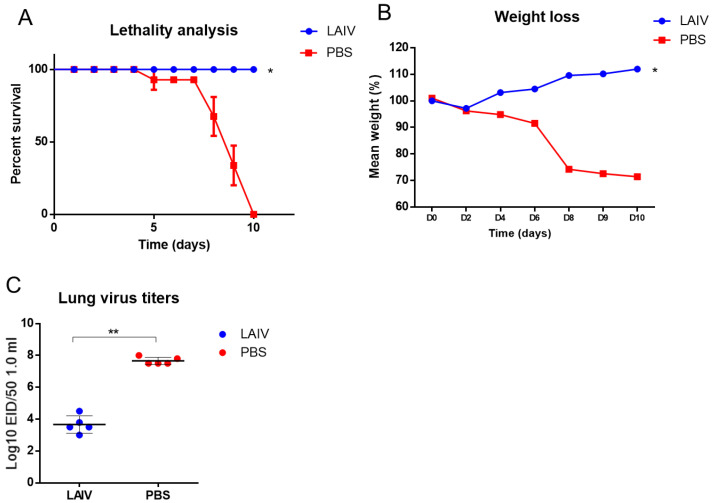
Challenge using 10 MLD50 of A/South Africa/3626/13(H1N1)pdm09 influenza virus on the 6th day, following A/17/South Africa/2013/01 immunization. * *p* < 0.05, ** *p* < 0.01 compared to PBS group. (**A**) Survival proportions (*n* = 10 per group). (**B**) Weight loss (*n* = 10). (**C**) Infectious-virus isolation from the lungs, 48 h after virus challenge (*n* = 5).

Then, we investigated how LAIV would protect against homologous infection in the early stages. To perform this, we conducted the immunization with the A/17/South Africa/2013/01(H1N1)pdm09 influenza virus and also infected immune mice with a lethal dose of A/South Africa/3626/13(H1N1)pdm09. When the immunization was performed with the LAIV based on pandemic influenza virus A/H1N1pdm09, the mice were 100% protected against infection with the homologous infecting virus (Figure 2A), while, in the group of non-immunized animals, all animals died by the 10th day following infection. This was also accompanied by a significant decrease in the content of infectious virus in the lungs (Figure 2C) and, in addition, the weight loss of the immunized animals following infection did not exceed 4%, in contrast to the non-immunized animals (Figure 2B).

Thus, it has been shown that LAIV can provide homologous and heterologous protection against influenza infection as early as the first week following vaccination.

### 3.2. Early Cytokine Production in THP-1 Cell Line

**Figure 3:**
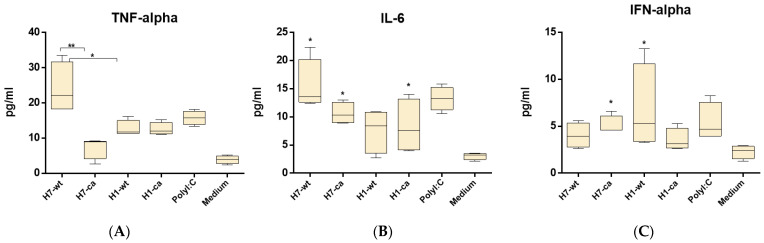
Early cytokine production in THP-1 cell culture, ELISA. H7-wt—A/Mallard Netherlands/12/2000(H7N3); H7-ca—A/17/Mallard Netherlands/00/95(H7N3); H1-wt—A/South Africa/3626/2013(H1N1)pdm09; H1-ca—A/17/South Africa/2013/01(H1N1)pdm09. Wells containing only RPMI medium were used as a negative control. The data of two independent experiments performed in two repetitions each are presented. (**A**) * *p* < 0.05, ** *p* < 0.01. (**B**) * *p* < 0.05 compared to negative-control wells. (**C**) * *p* < 0.05 compared to negative-control wells.

When introducing vaccine viruses and parental “wild-type” influenza strains into THP-1 cell line, the A/Mallard Netherlands/12/2000(H7N3) strain caused a notable increase in TNF-α and IL-6 levels (Figure 3A,B). This increase was significantly higher than the increase produced by the A/H7N3 vaccine virus or both A/H1N1 pdm09 viruses. Most of all, IFN-α was stimulated following the introduction of the “wild-type” A/H1N1 pdm09 virus, and the A/H7N3 vaccine virus caused the induction of IFN-α even in levels higher than the “wild-type” A/H7N3 virus the “wild-type” A/H1N1 pdm09 virus, and the A/H7N3 vaccine virus caused the induction of IFN-α even in levels higher than the wild-type A/H7N3 virus (Figure 3C).

### 3.3. Evaluation of the Pneumococcal Invasion following Influenza Infection

**Figure 4:**
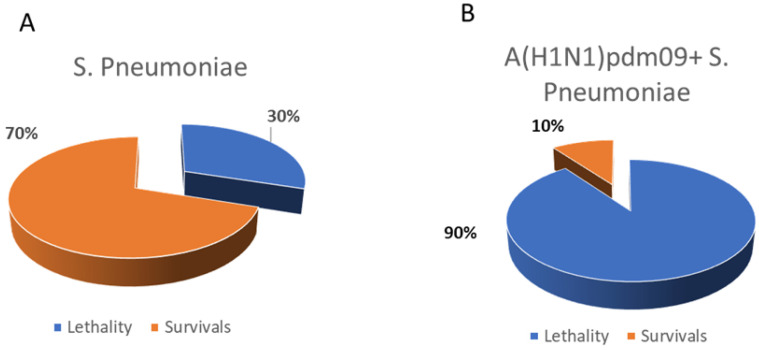
Post-influenza pneumococcal infection of the intact mice. (**A**) Challenge using 5 × 10^4^ CFU of *S. pneumoniae* (*n* = 10). (**B**) Challenge with pandemic influenza strain A/South Africa/3626/2013 (H1N1)pdm09, followed by *S. pneumoniae* infection 3 days apart.

Figure 4 shows the proportions of lethality in groups of non-vaccinated naïve mice infected with *S. pneumoniae*, compared to pneumococcal invasion conducted against the background of infection with a non-lethal dose of A/South Africa/3626/2013(H1N1)pdm09 influenza virus (~0.4 log10 EID_50_).

It was shown that, when mice were infected with only *S. pneumoniae*, the lethality did not exceed 30% (Figure 4A). Additionally, with a previous infection with the influenza virus of the pandemic subtype, the mortality rate reached 90% (Figure 4B).

Thus, the previous influenza challenge significantly worsened the course of post-influenza pneumococcal infection. In this regard, we decided to study how LAIV affects the development of post-influenza pneumococcal infection.

### 3.4. The Effect of Immunization with LAIV on the Resistance to Secondary Post-Influenza Pneumococcal Pneumonia

**Figure 5:**
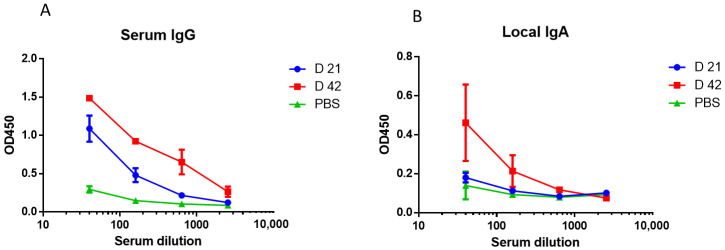
Antibody response to A/South Africa/3626/13(H1N1)pdm09 influenza virus estimated by ELISA, three weeks on day 21 after 1st and 2nd doses of trivalent LAIV containing A/17/New York/15/5364(H1N1)pdm09 (*n* = 6). (**A**) Serum IgG. (**B**) Local IgA.

We conducted a two-dose immunization with trivalent LAIV 2017–2018 years of formulation containing A/17/New York/15/5364 (H1N1)pdm09, A/17/Hong Kong/14/8296(H3N2) and B/60/Brisbane/08/83 vaccine strains [19]. The determination of serum and local antibodies to A/South Africa/3626/13(H1N1)pdm09 influenza virus showed that, following the second vaccine dose, the levels of antibodies to the challenge virus significantly increased compared to the first immunization (Figure 5).

**Figure 6:**
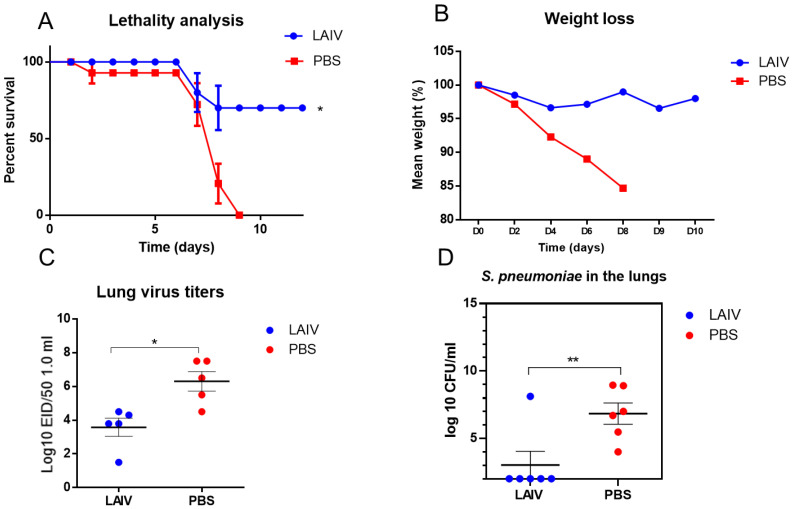
Protection against A/South Africa/3626/2013(H1N1) pdm09 influenza challenge, followed by *S. pneumoniae* infection 3 days apart. * *p* < 0.05, ** *p* < 0.01 compared to PBS group. The mice were immunized twice using trivalent LAIV 2017–2018 years of formulation. (**A**) The survival rate (*n* = 10 in group). (**B**) Weight loss (*n* = 10 in group). (**C**) Infectious-virus isolation from the lungs 48 h after virus challenge (*n* = 5). (**D**) *S. Pneumoniae* levels in the lungs of mice 48 h after bacterial superinfection.

It was shown that immunization with trivalent LAIV significantly protected mice from sequential influenza A/H1N1pdm09 and pneumococcal infections, when pneumococcal infection was induced on day three after primary influenza infection (Figure 6).

This was expressed by the fact that mortality decreased to 30%, and weight loss decreased as well (Figure 6A,B). Vaccination with LAIV not only led to a decrease in the levels of the infecting virus in the lungs (Figure 6C), but also reduced the pneumococcal contamination of the lungs (Figure 6C).

**Figure 7:**
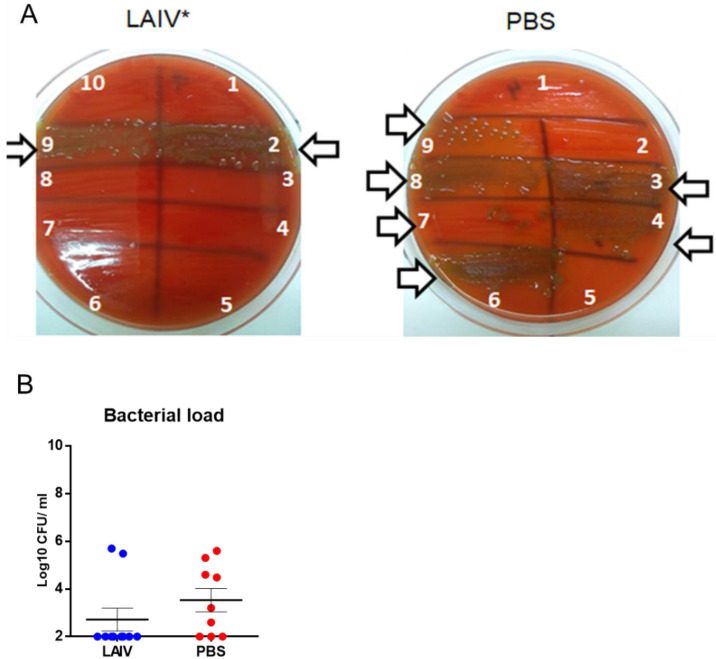
The results of the assessment of bacteriemia in infected animals on day 5, following bacterial superinfection. The mice were challenged using A/South Africa/3626/2013(H1N1) pdm09 influenza virus followed by *S. pneumoniae* infection 3 days apart. (**A**) Counting the number of bacteria in the blood of LAIV-immunized and mock-immunized mice. Arrows indicate pneumococcal colonies. The white numbers denote each of the mice. * *p* = 0.02 compared to PBS group (Fisher’s exact test). (**B**) Assessment of average bacterial-load levels in the blood. Blue and red dots mean individual bacterial titers (Log10 CFU/mL).

Subsequently, we tried to assess the level of bacteremia in animals infected with pneumococci against the background of a viral infection. For this, blood was obtained from the mice from the submandibular vein on the sixth day following pneumococcal infection and inoculated on “blood” agar (Figure 7).

It was shown that, following immunization with LAIV, pneumococci were isolated from only two mice, while bacteremia was observed in six out of nine non-immunized animals (Figure 7A). These differences were statistically significant (*p* = 0.02). The mean shedding of bacteria from the blood of immunized animals was lower than that of non-immunized animals, although the differences were not statistically significant (Figure 7B).

## 4. Discussion

The use of intranasal LAIVs appears to be very important during the current epidemic situation. First, it is very important to vaccinate against influenza during the period of the rise in SARS-CoV-2, since it has been shown that co-infection with influenza significantly worsens the course of COVID-19 [20]. Secondly, the use of intranasal LAIV allows the vaccination of large population groups. Finally, only LAIVs are able to create IgA-mediated local immunity in the upper respiratory tract and interrupt the transmission of an infectious virus [21], in contrast to inactivated vaccines, as has been repeatedly shown in large-scale LAIV studies conducted in the 1980s–1990s [22,23]. One of the explanations for this effect may be the induction of type-I interferon with the introduction of LAIV. Interestingly, vaccine viruses may cause even more substantial interferon production than “wild-type” viruses. It was previously shown that the vaccine candidate based on A/PR8/34(H1N1) with a deleted NS gene has been shown to stimulate the production of interferon in chicken embryos higher than the wild A/PR8 virus, and can provide protection before the development of specific adaptive immunity [24]. In our previous mouse study, we evaluated early protection provided by vaccination with a combination of LAIV and recombinant bacterial peptides against homologous and heterologous influenza virus challenges. Improved protection was followed by a decrease in the content of infectious viruses in the lungs, which correlated with an increase in the expression of IFN-α in the lungs [25].

In the current study, intranasal immunization using potentially pandemic and pandemic-LAIV-protected mice from a challenge with pandemic influenza virus A/South Africa/3626/2013(H1N1)pdm09. To assess the factors of innate immunity in response to influenza viruses, the levels of TNF-α, IL-6, and IFN-α produced by THP-1 cells was studied after inoculation with vaccine influenza A viruses or “wild-type” viruses. Macrophages significantly contributed to the early cytokine production during influenza infection. Previous studies have shown that, at the onset of infection, macrophages produce higher levels of IFN-α, as well as chemokines involved in the migration of leukocytes from the bloodstream to the inflammatory site, compared to epithelial cells. Several previous studies showed that human macrophages, following exposure to both live or inactivated influenza A viruses, produced expressed quantities of type-I interferons 24 h following inoculation [26]. It is known that early cytokines TNF-α and IL-6 are involved in the development of inflammation at the site of infection and cause immune activation and recruitment of macrophages. At the same time, these cytokines are responsible for the acute symptoms of influenza and pathology associated with influenza infection, as they are strong pyrogens and inducers of eicosanoid production [27,28]. In our study, in THP-1 cells, it has been shown that only the “wild-type” A/H7N3 virus of avian origin caused a strong release of TNF-α and IL-6, and vaccine viruses only caused a moderate increase in the levels of these cytokines. On the other hand, the vaccine virus A/H7N3 induced an increase in the level of IFN-α, which may play a role in providing early protection by the heterologous influenza virus.

The study of innate immunity factors during immunization with newly developed vaccines is important in light of the fact that sometimes, for example, in old age, adaptive T- and B-immune response may partially be replaced by innate function [29]. In addition, upregulation genes for cytokines and their receptors at an early stage of vaccination may be markers of subsequent antibody response and its duration [30,31].

Influenza virus infection makes the airways more susceptible to bacterial infections, which often cause severe secondary post-influenza complications, such as bronchitis and pneumonia [32]. Secondary bacterial complications from influenza are of great socio-economic importance. In our study, in mice, we demonstrated that pneumococcal infection following infection with the influenza virus caused significantly higher mortality compared to bacterial infection alone. Vaccination against influenza can significantly reduce the risk of secondary bacterial complications following influenza infection [33,34]. In the present mouse study, it was shown that seasonal trivalent LAIV in mice not only reduced mortality during post-influenza pneumococcal infection, reducing viral reproduction in the lungs, but also lowered the content of *S. pneumoniae* in the lungs and partially prevented systemic pneumococcal infection.

Study limitations: Although we did not experimentally study the effect of LAIV on the subsequent bacterial contamination of the respiratory tract in this study, it was previously shown that immunization with influenza vaccines mitigates the subsequent development of post-influenza bacterial complications [31]. Additionally, our colleagues have shown that the introduction of LAIV did not increase the sensitivity of mice to bacterial contamination [35].

## 5. Conclusions

Monovalent LAIV provided early protection against homologous and heterologous influenza infections. Seasonal trivalent LAIV improved the course of post-influenza bacterial infections. These data suggest that intranasal administration of LAIV may be useful during the period of circulation not only of influenza viruses, but also of other pathogens of acute respiratory infections.

## Figures and Tables

**Table 1:** Vaccine influenza viruses used to immunize mice.

Vaccine Strain	Subtype/Lineage	‘Wild-Type’ Parent Virus
A/17/Mallard Netherlands/00/95	(H7N3)	A/Mallard Netherlands/12/2000
A/17/South Africa/2013/01	(H1N1)pdm09	A/South Africa/3626/2013
A/17/New York/15/5364	A/17/New York/15/5364	A/New York/61/15
A/17/Hong Kong/14/8296	(H3N2)	Hong Kong/4801/14
B/60/Brisbane/08/83	B/Victoria	Brisbane/60/08

## Data Availability

All data is contained within article.
